# A Bluetooth-Enabled Device for Real-Time Detection of Sitting, Standing, and Walking: Cross-Sectional Validation Study

**DOI:** 10.2196/47157

**Published:** 2024-01-24

**Authors:** Reza Daryabeygi-Khotbehsara, Jonathan C Rawstorn, David W Dunstan, Sheikh Mohammed Shariful Islam, Mohamed Abdelrazek, Abbas Z Kouzani, Poojith Thummala, Jenna McVicar, Ralph Maddison

**Affiliations:** 1 Institute for Physical Activity and Nutrition, School of Exercise and Nutrition Sciences Deakin University Melbourne Burwood Australia; 2 Baker-Deakin Department of Lifestyle and Diabetes Melbourne Burwood Australia; 3 School of Information Technology Deakin University Melbourne Burwood Australia; 4 School of Engineering Deakin University Geelong Australia

**Keywords:** activity tracker, algorithms, deep neural network, machine learning, real-time data, Sedentary behaviOR Detector, sedentary behavior, SORD, standing, validation, walking, wearables

## Abstract

**Background:**

This study assesses the accuracy of a Bluetooth-enabled prototype activity tracker called the Sedentary behaviOR Detector (SORD) device in identifying sedentary, standing, and walking behaviors in a group of adult participants.

**Objective:**

The primary objective of this study was to determine the criterion and convergent validity of SORD against direct observation and activPAL.

**Methods:**

A total of 15 healthy adults wore SORD and activPAL devices on their thighs while engaging in activities (lying, reclining, sitting, standing, and walking). Direct observation was facilitated with cameras. Algorithms were developed using the Python programming language. The Bland-Altman method was used to assess the level of agreement.

**Results:**

Overall, 1 model generated a low level of bias and high precision for SORD. In this model, accuracy, sensitivity, and specificity were all above 0.95 for detecting sitting, reclining, standing, and walking. Bland-Altman results showed that mean biases between SORD and direct observation were 0.3% for sitting and reclining (limits of agreement [LoA]=–0.3% to 0.9%), 1.19% for standing (LoA=–1.5% to 3.42%), and –4.71% for walking (LoA=–9.26% to –0.16%). The mean biases between SORD and activPAL were –3.45% for sitting and reclining (LoA=–11.59% to 4.68%), 7.45% for standing (LoA=–5.04% to 19.95%), and –5.40% for walking (LoA=–11.44% to 0.64%).

**Conclusions:**

Results suggest that SORD is a valid device for detecting sitting, standing, and walking, which was demonstrated by excellent accuracy compared to direct observation. SORD offers promise for future inclusion in theory-based, real-time, and adaptive interventions to encourage physical activity and reduce sedentary behavior.

## Introduction

Sedentary behavior (SB) is defined as “any waking behavior characterized by an energy expenditure of less than 1.5 metabolic equivalents while in a sitting, reclining, or lying posture” [1,2]. SB is an independent risk factor for many noncommunicable diseases, with the risk being most pronounced in those who are also physically inactive (ie, not meeting physical activity [PA] guidelines) [3-5]. Reducing SB for all people, including those who are physically active, can assist in producing health benefits [6]. Interrupting SB with standing or light or moderate intensity PA can improve chronic risk factors including glucose homeostasis, insulin sensitivity, blood lipid concentrations, and diastolic blood pressure [7-10]. Recent World Health Organization guidelines on PA and SB explicitly state the importance of reducing sedentary time in addition to promoting PA for adults and older adults, including those with chronic conditions [11]. This has subsequently led to the development of interventions targeting SB reduction, although interventions to date have been compromised by the lack of a tool that can capture SB accurately and in real time. Accurate measurement of sitting, standing, and walking in real time will enable the design of interventions that can adapt to changes in the activity state and can be delivered at times when an individual is most responsive to the intervention, therefore maximizing the potential opportunity for reducing SB and increasing PA [12].

To date, the majority of interventions to reduce SB and promote PA have relied on subjective measurement of these behaviors, which are subject to self-report bias [13,14] and may underestimate daily sitting time by up to 2 hours compared with objective measurement [15]. Few activity trackers, including research-grade (eg, activPAL) and commercial (eg, GENEactive and Fitbit One), measure sedentary time with reasonable precision [16-22], but they are not optimal for SB change interventions [23]. The 2 main issues involve technical difficulty in using support software for real-time interventions and concerns about device accuracy in distinguishing postural states (sitting, standing, etc) [24,25]. Most activity trackers use similar technologies, including accelerometers, magnetometers, and gyroscopes, to detect posture and activity [26]. However, the placement of devices on the body can considerably influence accuracy [27]. Commercial wrist-worn devices such as the Garmin Vivofit are unable to detect sit-to-stand transition [23,28]. Other thigh-worn devices, such as activPAL and SitFIT, are capable of detecting sitting and standing due to their horizontal placement [23]. In terms of behavioral intervention, activPAL does not offer any real-time prompts or feedback to participants [29]. The SitFit device provides real-time feedback to the user, and its accuracy, although acceptable, was lower when compared to the activPAL, which is considered the preferred device for research purposes [30]. However, SitFit is pocket-worn, which limits its use for those not wearing suitable clothing (eg, trousers) or garments without pockets (eg, dresses) [30]. More importantly, SitFit does not distinguish standing from walking [31] and therefore cannot be used to assess standing as a unique outcome both for real-time and adaptive interventions. It should be noted that these devices (SitFit and Fitbit One) are no longer available on the market and were included in our discussion to provide historical context and illustrate the evolution of activity-tracking technology. Evidence on the positive impact that standing may have on health outcomes in different population groups is emerging from short-term and small-scale studies [32,33], although real-time assessment and behavior change interventions are missing. This, in turn, suggests a need for a platform to momentarily evaluate both sedentary and standing outcomes to study their exclusive health effects and intervene accordingly.

In summary, despite the presence of activity tracker devices, few have included evidence- and theory-based interventions or strategies to promote PA and reduce SB (eg, self-monitoring and goal setting), and the use of some other devices is restricted due to a lack of real-time assessment of outcomes (eg, standing). In response, we designed and developed a new wearable platform called “Sedentary behaviOR Detector” (SORD), which collects real-time sedentary data, including lying, reclining, sitting, and standing, as well as walking activity time. Therefore, this study aimed to assess the validity of the SORD device in detecting sedentary and walking activities among adult participants.

## Methods

### Overview

A cross-sectional, laboratory-based study was conducted to assess the criterion validity (SORD vs direct observation) and convergent validity (SORD vs activPAL). Adults were recruited to take part in this laboratory-based study through print and email advertisements at a university campus. Adults aged 18 years or older, without gait abnormalities, able to walk on a treadmill easily, with no skin sensitivity to plasters or tapes, and able to communicate in English were included.

Upon arrival, participants completed a demographic questionnaire including age, sex, ethnicity, job status, marital status, education, and the Physical Activity Readiness Questionnaire [34] for safe exercise. Anthropometric measures, including height to the nearest 0.1 cm and weight to the nearest 0.1 kg, were taken using a stadiometer (Seca 213) and Tanita scale (Tanita Innerscan 50), respectively.

Participants were given a printed activity protocol to help familiarize them with the required activities and the order in which they were to be performed. Textbox 1 presents a range of different states of activities included in the study protocol to mimic typical postures that may be encountered during everyday life.

Hypoallergenic retention dressing tape (Hypafix) was used to attach the SORD and activPAL devices on the midline of the right thigh. Participants were then instructed to engage in a combination of activities in the order of sitting, reclining, sitting, standing, walking, standing, sitting, lying, and walking on a treadmill. Each activity variation lasted for a minimum of 2 minutes and a maximum of 3 minutes and 30 seconds, except walking, which involved participants walking at their regular walking pace along a 10-m-long path. Participants had 2 minutes of optional resting to break up the activities if needed. Ground truth, or the true time spent on each of the activities, was measured by a researcher with the help of a video camera for direct observation.

**Details of the Sedentary behaviOR Detector phase 1 activities.**

**Lying**

- Face up, on the right shoulder, face down, or on the left shoulder

**Reclining**

- Normal (135 slope chair), left leg over right, or right leg over left

**Sitting**

- Upright, ankle-on-knee (left-right and right-left), right foot move, left foot move, both feet move, elbows on legs, or sitting with outstretched legs

**Standing**

- Stand normal, casual standing (more weight on the right foot), casual standing (more weight on the left foot), right shoulder on the wall, or left shoulder on the wall

**Walking**

- Normal on level, on treadmill at 4 km/h, or on treadmill at 6 km/h

### Sedentary behaviOR Detector

#### Overview

**Figure 1:**
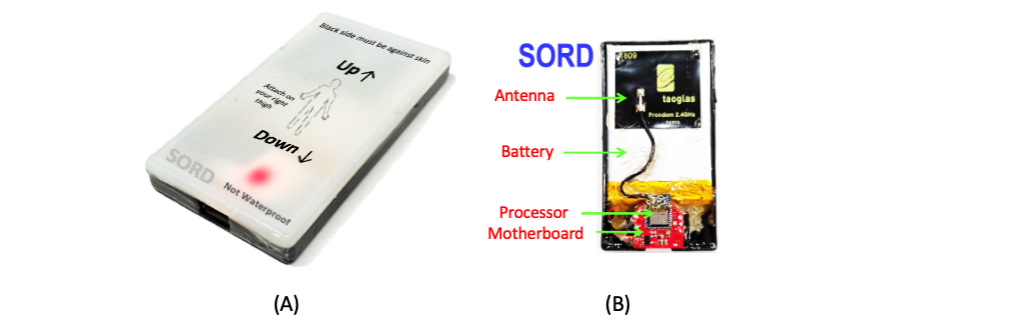
(A) Sedentary behaviOR Detector (SORD). (B) Internal components of the SORD device. The SORD is a small device with the following dimensions: 0.9 mm (height), 37 mm (width), and 68 mm (length). It is also lightweight, with a weight of 23.5 g. The device can operate for about 45 hours on a single charge. The SORD device measures 3-axis orientation using the accelerometer that gives acceleration signals for 3 axes, the gyroscope that provides rotation along 3 axes, and the magnetometer that gives motion in the magnetic field in 3 axes. It hosts an embedded C firmware that continuously reads from the sensors, records their data at 25-Hz frequency, preprocesses the data, and transmits the data wirelessly. No initialization is required for the SORD device, as the data are captured and transmitted through the 2.4-GHz Bluetooth Low Energy 5.0 transceiver in real time.

The SORD is a wearable electronic device (Figure 1A) that collects and provides real-time data associated with sitting, reclining, lying, and PA. Data provided by the device can be used to separate sitting versus standing versus ambulation. To separate sitting time from lying time, 2 same devices will be attached to 2 different locations of the body.

The SORD device includes a number of internal components (Figure 1B): a low-power processor and transceiver, inertial measurement unit, voltage regulator, battery charger, battery, antenna, micro-USB connector, LEDs, motherboard, and an enclosure. These components have been described below.

#### Processor and Transceiver

The ATSAMB11-ZR210CA is used that includes a low-power ARM Cortex M0 32-bit processor, 128 KB of RAM, 128 KB of stacked flash memory, a 2.4 GHz Bluetooth Low Energy 5.0 transceiver and modem, a power management unit, a ceramic high-gain antenna, and a printed circuit board with a small footprint.

#### Inertial Measurement Unit

The BNO055 is used that includes a single-chip integrated circuit incorporating an intelligent inertial measurement unit with a triaxial 14-bit accelerometer, a triaxial 14-bit gyroscope, a triaxial geomagnetic sensor, an I2C communication interface, and an ARM Cortex M0+ 32-bit processor executing a sensors data fusion algorithm.

#### Voltage Regulator

The XC9264B755MR-G is used which includes a synchronous step-down DC/DC voltage regulator. It operates within the voltage range of 3-18 V and provides a 500 mA output current. It has a selectable switching frequency of 500 kHz, 1.2 MHz, or 2.2 MHz. It also features overcurrent protection as well as thermal shutdown.

#### Battery Charger

The BQ25101YFPR is used which includes a linear Li-Ion and Li-Pol battery charger with a very small footprint. It has a single power output that charges a battery in 3 steps: conditioning, constant current, and constant voltage. The junction temperature of the device is monitored to control the charge current.

#### Micro-USB Connector

A micro-USB connector is used for programming the processor and also for establishing serial communications as well as charging the onboard battery.

#### LEDs

A total of 2 multicolor LEDs are used to illuminate different functional states of the device to the user.

#### Battery

A 3.7-V, 700-mAh, 303759 Lithium Polymer rechargeable battery is used. Its height, width, and length are 3 mm, 37 mm, and 59 mm, respectively, and its weight is 14 g.

#### Antenna

A Freedom 2.4-GHz flex circuit PCB antenna is used.

#### Motherboard

A printed circuit motherboard is designed and fabricated to host all the electronic components of the SORD device.

#### Enclosure

A small enclosure for the SORD device is designed and 3D printed. It hosts all the components of the device.

### ActivPAL

ActivPAL is a thigh-worn triaxial accelerometer that classifies an individual’s activity into periods of time spent sedentary (lying or sitting), standing, and walking, as well as the number of steps and stepping speed [29,35]. ActivPAL devices were initialized before the data collection and date-time stamped 1-second epoch files were used for comparative analysis.

### Direct Observation

True time spent engaging in activities was logged by a trained researcher (RDK). This was assisted by a video camera positioned in the room and checked by another researcher (JM). If there was any discrepancy, RDK and JM reviewed the camera data together to achieve consensus. No formal intra- or interrater reliability was conducted.

### Data Handling and Analysis

SORD data were transmitted to a computer through Bluetooth Low Energy. A program was developed in MATLAB (MathWorks) and run on a Microsoft Windows (Microsoft Corp)–based computer to receive data from the SORD devices in real time and store it into a Microsoft Excel (Microsoft Corp) file. The program starts by initializing relevant variables and a communications port, creates a file name based on the current date and time, continuously receives data from the SORD devices, and stores the incoming data in the Microsoft Excel file in real time. Each data packet received from the SORD devices includes values obtained from the onboard sensors at the current time. For a data packet, the following information is then stored in the file in real time: date, time, angle, accelX, accelY, accelZ, gyroX, gyroY, gyroZ,magX, magY, magZ, and battery voltage. To avoid potential Bluetooth transmission package loss, this study used the time-stamp data from the SORD device instead of the computer receiver (ie, the sending time stamp rather than the receiving time stamp). Thus, we had computer receiver and accelerometer data, along with their timestamps. Based on the real sampling rate, the computer calculated the time stamp difference between each data point (ΔT). In this research, the number of missing data points was defined by missing = ΔT/(1/25Hz) – 1. The values of these data points were filled by the average of the 2 data points before and after the missing data points (eg, Vi[missing] = [Vi – 1 + Vi + 1]/2). Before sending data to the server for inference, the phone app waits until all required data have been received (processing buffer length). Using the VANE (standard) classification algorithms, activPAL data were processed and collected using proprietary software (activPAL Professional Research Edition, PAL Technologies). The software-generated event file was used. This file contains a chronological list of all episodes of sedentary, standing, and stepping (ie, walking) activities recorded at 1-second intervals. The frequency of the recorded signals from SORD was subsequently reduced to 1 Hz (ie, 1-second epochs) for comparative analysis. This reduction in frequency simplifies data processing and facilitates direct comparison with activPAL, which was also sampled at 1 Hz. Furthermore, outliers or irregular data points were identified and removed. Once individual data sets were cleaned, they were combined for subsequent comparative analysis. The combining process involved aligning the data sets temporally so that corresponding data points from both devices were synchronized for direct comparison.

Due to multiple limitations, we did not use the available open-source activity recognition algorithms. These limitations include (1) inconsistency in the data format and ranges, (2) differences in the frequency of raw data assumed by these algorithms compared to SORD (which is 28 measurements per second), and (3) the variations of activities considered by these algorithms were not exactly the same as the ones we wanted to address in this research at this stage and in the future. Thus, we developed the data engineering and activity recognition models. Although ensemble learning techniques outperform deep learning, they demand higher computation resources and have longer processing latency [36,37]. Therefore, for practical reasons and real-world applications of SORD, this study used deep neural network models—a combination of convolutional neural network and recurrent neural network—to develop algorithms. Deep neural network can learn features automatically from the raw data, therefore performing better than statistical and basic machine learning methods, and they are suitable for recognizing complex activities [38].

A data scientist developed deep learning algorithms to classify activity type and postural states from preprocessed motion sensor data using the Python programming language [39]. First, machine learning classifiers were developed, trained, and tested for the SORD device. A dynamic sliding window approach was used for machine learning [40], where each window was related to a particular activity and multiple variables were examined within each window to identify patterns. When a particular activity was detected in the sensor readings, features were extracted to classify activities between the previous one and the current one (further details are provided below). Then, criterion validity (against direct observation) and convergent validity (against activPAL micro) were evaluated. Using Python, the Bland-Altman method was used to assess the level of agreement between SORD and each reference measure (criterion agreement=directly observed time and convergent agreement=activPAL). Mean difference represents the systematic bias, and the limits of agreement (LoA) show the range of agreement between SORD and reference methods, where a positive value indicates underestimation and a negative value indicates overestimation by SORD. For all activity states, we predefined the acceptable LoA between ±10%.

### Classification Algorithms

A single data set included SORD, activPAL, and direct observation data for 1 participant. Deep learning was used to randomly select 6 data sets for training, 1 for validation, and 7 for testing. In the training set, similar patterns were identified for the previous 35 data points to specify an activity. Confusion matrices were used to visualize the model’s performance. In a confusion matrix, each row represents the instances in the predicted activity, and each column represents the instances in the actual activity.

### Ethical Considerations

Ethics approval was granted by the Deakin University Human Research Ethics Committee’s Human Ethics Advisory Group (HEAG-H 109_2019). All participants provided written informed consent. All research data were anonymized before cleaning and analysis. Participants were remunerated with an Aus $20 (US $14) gift voucher.

## Results

### Overview

In total, 15 adults (12 female adults) aged between 20 and 62 years completed the experimental study. Table 1 presents the demographic characteristics of the participants.

**Table 1:** Demographic information of study participants.

Variables			Values
**Age (years)**			
	Mean (SD)	35.2 (11.6)	
	Range	20-62	
**Weight (kg)**			
	Mean (SD)	70.4 (10.5)	
	Range	55.2-84.8	
**Height (cm)**			
	Mean (SD)	168.1 (9.6)	
	Range	147.0-186.5	
**BMI (kg/m^2^)**			
	Mean (SD)	24.9 (3.0)	
	Range	20.1-29.4	
**Sex, n (%)**			
	Female	12 (80)	
	Male	3 (20)	
**Ethnicity, n (%)**			
	Australian	4 (27)	
	European	6 (40)	
	Asian	4 (27)	
	South American	1 (7)	
**Education level, n (%)**			
	Degree higher than bachelor’s (bachelor’s with honors, masters, or PhD)	7 (47)	
	Bachelor’s degree	5 (33)	
	Technical and further education or university course below a bachelor’s degree	2 (13)	
	Other school qualifications (eg, overseas school, Cambridge examination, or A level)	1 (7)	
**Job status, n (%)**			
	Full-time salary or wage earner	6 (40)	
	Part-time salary or wage earner	2 (13)	
	Student	7 (47)	
**Marital status, n (%)**			
	Married or living with a partner	8 (53)	
	Single or never married	1 (7)	
	Separated, divorced, or widowed	6 (40)	

### Deep Learning Results

**Figure 2:**
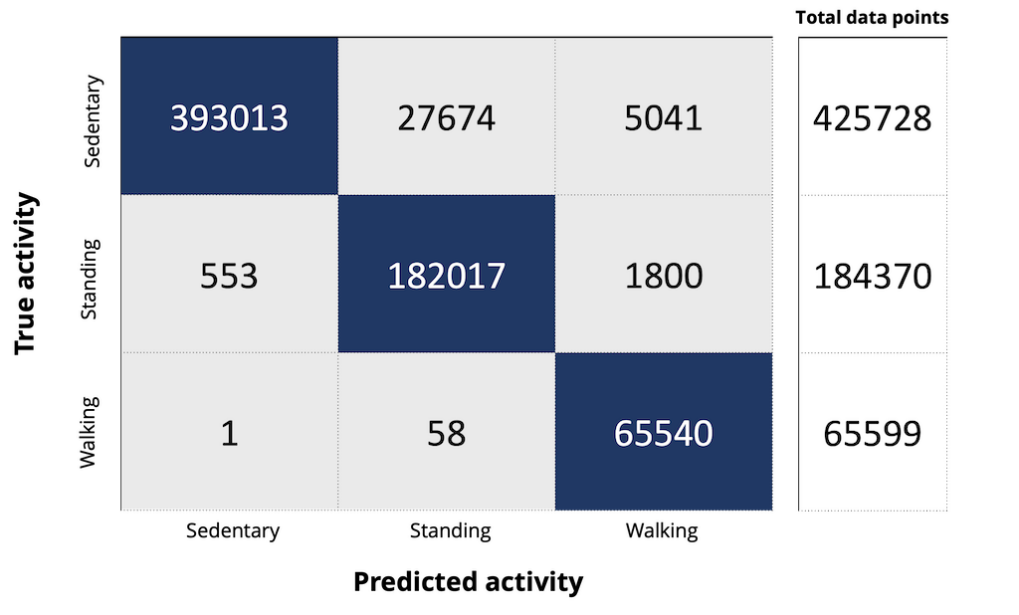
Confusion matrix for model 1 classification algorithms. Sedentary (lying, sitting, and reclining), standing, and walking were included in the model.

A total of 4 models were presented for SORD. Model 1 classified 3 activities, including sedentary (lying, reclining, or sitting), standing, and walking separately. As illustrated in Figure 2, model accuracy, sensitivity, and specificity for detecting sedentary time were 0.92, 0.99, and 0.87; for standing, they were 0.95, 1.00, and 0.91; and for walking, they were 0.96, 0.92, and 1.00, respectively.

**Figure 3:**
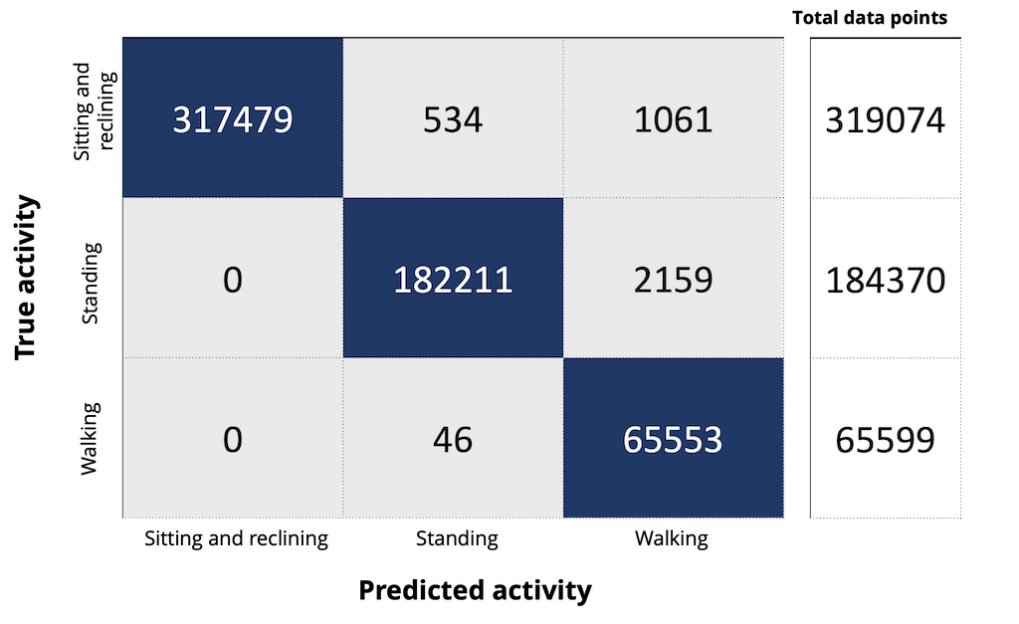
Confusion matrix for model 2 classification algorithms. “Sitting and reclining,” standing, and walking were included in the model.

Model 2 included 4 activities: sitting, reclining, standing, and walking; lying was excluded (ie, lying moments observed by video camera were omitted from the data set). As illustrated in Figure 3, model accuracy, sensitivity, and specificity for detecting sitting and reclining were 1.00, 1.00, and 1.00; for standing, they were 0.99, 0.99, and 1.00; and for walking, they were 0.98, 1.00, and 0.95, respectively.

Model 3 included 3 activities: sitting, standing, and walking; reclining and lying were excluded. Respectively, model accuracy, sensitivity, and specificity for detecting sitting were 0.97, 1.00, and 0.94; for detecting standing, they were 0.95, 0.91, and 1.00; and for walking, they were 0.98, 1.00, and 0.97 (Multimedia Appendix 1).

Model 4 included all 5 activities: lying, sitting, reclining, standing, and walking. Respectively, model accuracy, sensitivity, and specificity for detecting lying were 0.70, 0.54, and 1.00; for sitting and reclining, they were 0.85, 1.00, and 0.75; for standing, they were 0.75, 0.63, and 0.93; and for walking, they were 0.99, 1.00, and 0.98 (Multimedia Appendix 2).

### Agreement

**Figure 4:**
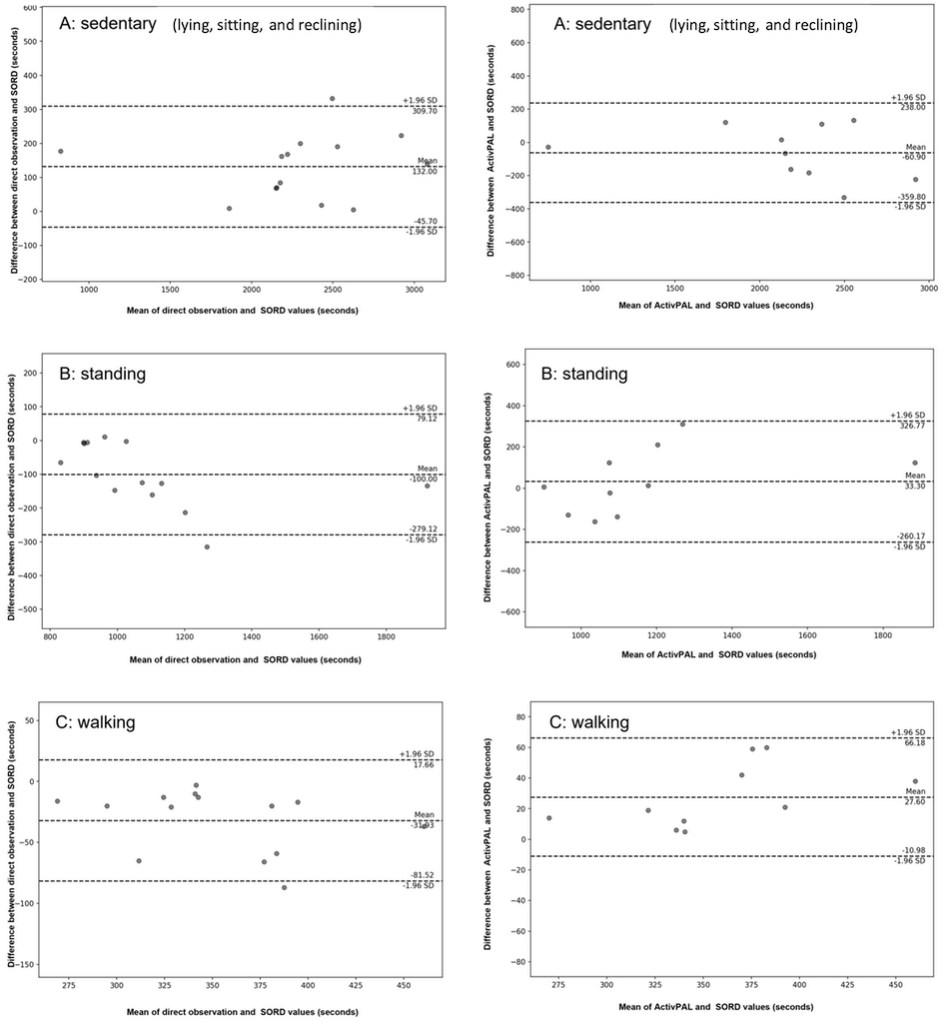
Bland-Altman plot comparing seconds of sedentary behavior, standing, and walking between direct observation and activPAL against the Sedentary behaviOR Detector (SORD) activity tracker (model 1).

**Figure 5:**
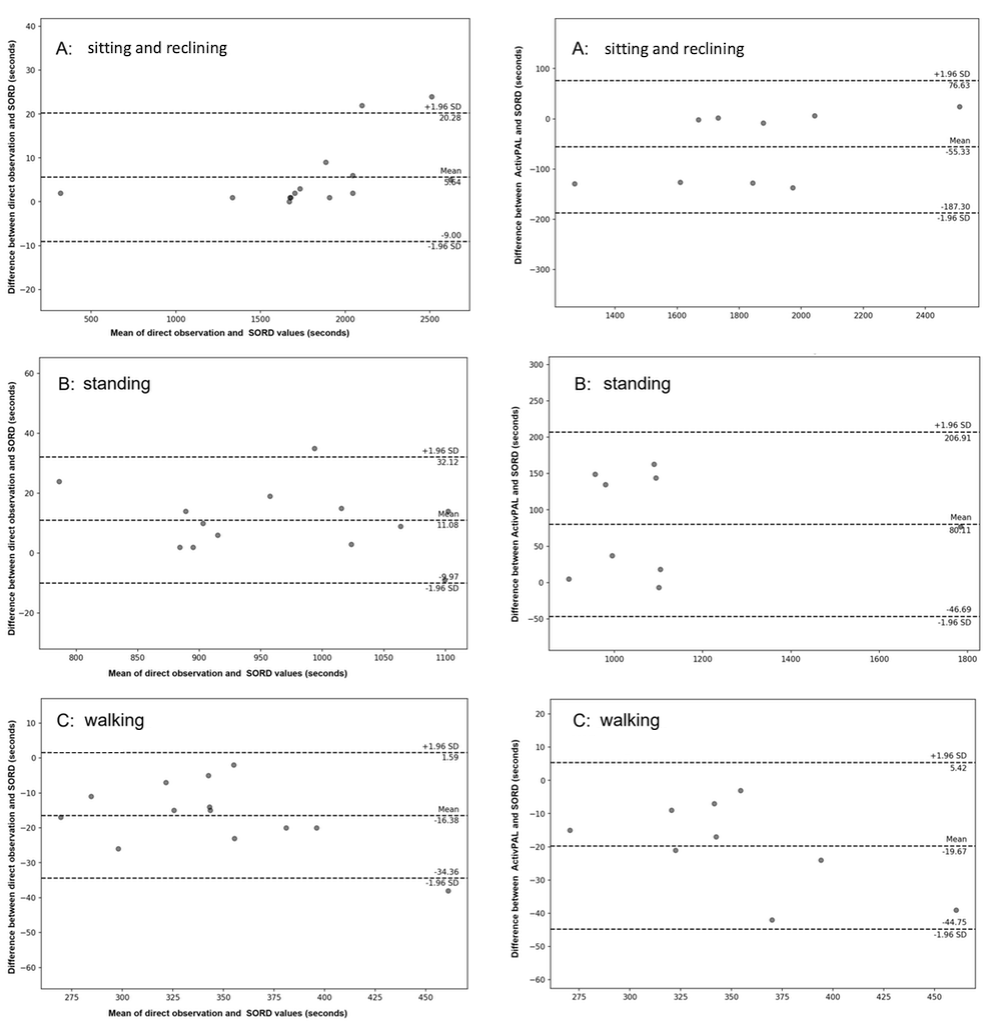
Bland-Altman plot comparing seconds of “sitting and reclining,” standing, and walking between the direct observation and activPAL against the Sedentary behaviOR Detector (SORD) activity tracker.

For models 1 and 2, results of the Bland-Altman analysis comparing second-by-second data on sedentary, standing, and walking time between direct observation versus SORD and activPAL versus SORD are presented in Figures 4 and 5. Multimedia Appendices 3 and 4 illustrate Bland-Altman for the other models. Percentage values are presented in the text (see Figures 4 and 5 and Multimedia Appendices 3 and 4 for true values).

Mean differences (biases) between SORD model 1 and direct observation were 6.4% for sedentary (LoA=–4.3% to 17.1%), –8.7% for standing (LoA=–23.5% to 6.1%), and –8.9% for walking (LoA=–22.2% to 4.4%). Results of model 1 show wide limits, although the mean biases were below 10% for all activities. Relative to total activity durations, mean biases between SORD model 1 and activPAL were –2.5% for sedentary (LoA=–15.0% to 9.9%), 1.7% for standing (LoA=–23.3% to 26.9%), and 7.4% for walking (LoA=–2.3% to 17.1%). Results of model 1 comparing SORD to activPAL show wide limits.

Mean biases between SORD model 2 and direct observation were 0.3% for sitting and reclining (LoA=–0.3% to 0.9%), 1.19% for standing (LoA=–1.05% to 3.42%), and –4.71% for walking (LoA=–9.26% to –0.16%). Model 2 showed the narrowest LoA for “sitting and reclining,” standing, and walking, denoting excellent agreement with direct observation. All the mean biases were within ±10%. Relative to total activity durations, mean biases between SORD Model 2 and activPAL were –3.45% for sitting and reclining (LoA=–11.59% to 4.68%), 7.45% for standing (LoA=–5.04% to 19.95%), and –5.40% for walking (LoA=–11.44% to 0.64%). Results of model 2 comparing SORD to activPAL show a wider LoA, although mean biases are relatively low for “sitting and reclining” and walking.

Mean biases between SORD model 3 and direct observation were –6.4% for sitting (LoA=–18.6% to 5.7%), 12.4% for standing (LoA=–6.6% to 31.5%), and –4.9% for walking (LoA=–12.5% to 2.5%). Therefore, sitting and walking were overestimated, while standing was underestimated. The mean bias was acceptable for sitting and walking but not standing. A narrow LoA were observed for walking.

Mean biases between SORD model 4 and direct observation were 52.2% for lying (LoA=–6.9% to 111.4%), –32.0% for sitting and reclining (LoA=–78.2% to 14.1%), 48.8% for standing (LoA=–13.7% to 111.4%), and –2.2% for walking (LoA=–6.7% to 2.2%). Therefore, “sitting and reclining” and walking were underestimated while lying and standing were overestimated. Model 4 shows the broadest LoA for “sitting and reclining” and standing, while the narrowest LoA were observed for walking in this model.

## Discussion

This laboratory-based study assessed the criterion and convergent validity of a prototype activity tracker (ie, SORD). A high level of accuracy in detecting sitting, standing, and walking for the SORD device among adults was confirmed. Based on the Bland-Altman plots, high levels of agreement with direct observation demonstrated high criterion validity.

ActivPAL is a triaxial accelerometer that has been validated for detecting sitting, standing, and walking activity [29,41,42] and has been widely used in previous intervention studies [43-47]. However, a recent review found that activPAL has lower accuracy during fidgeting [48]. In this study, the agreement between SORD and activPAL was not ideal. The discrepancy observed might result from the inclusion of various fidgeting states. In addition, since activPAL does not enable real-time transmission of data to external devices or networks [29], it cannot be used for real-time or adaptive interventions. SitFit [30] is among the few devices that provide real-time feedback on SB. SitFit (PAL Technologies Ltd) is a pocket-worn device that requires appropriate clothing (eg, trousers with a front pocket), which is a barrier to its usability [30]. SitFit has an embedded screen to provide visual feedback to users and is also Bluetooth-enabled for connectivity to smartphones, tablets, and PCs. However, outputs generated by SitFit include sedentary time (sitting or lying), upright time, and step count [30]. The upright time includes both quiet standing and stepping [30], meaning that SitFit alone is not suitable for measuring standing as an outcome. Measuring standing and its variations (eg, fidgeting while standing) in real time will enable future intervention studies to identify distinct behavioral determinants of standing and to study its long-term clinical implications. As described in this study, SORD accurately measures sedentary (sitting and reclining), standing, and walking time. Other deep learning models (eg, model 4) examined whether the algorithms could distinguish lying from other sedentary states. A lower accuracy was observed for SORD in distinguishing lying from other sedentary activity states. Since the thigh is horizontal during lying posture, distinguishing sitting and lying postures with thigh-worn devices would be difficult. Methods that include rotational angle thresholds to determine the orientation of the thigh have been able to distinguish lying from sitting [49], even though these techniques require validation against direct observation to produce robust evidence.

A strength of this study is the inclusion of several variations of activity states (eg, sitting with outstretched legs, sitting while ankle-on-knee, and standing while shoulder on the wall), allowing more robust testing of the device accuracy and improving the generalizability of findings. For example, detecting standing as it appears in real-life situations and distinguishing from walking will enable the design of interventions measuring standing as a behavioral or clinical outcome. There are also limitations with this study, including the laboratory-based nature of the study. As with any laboratory-based experiment, it is possible that participants behave differently (eg, sit tall and neat and not as they would do normally). Moreover, a comparison between devices in terms of walking intensities was not conducted. This work is the first step in the validation of SORD, and longer-term studies in free-living environments would be necessary future steps to assess its practicality and accuracy under diverse conditions. The majority of participants in this study were female, and that might be considered a source of bias, that is, sex bias. However, evidence suggests that there are no significant differences between female individuals and male individuals in terms of posture, including sitting, standing, and walking [50]. Most participants were younger adults, and therefore the findings may not be generalizable to older adults. Investigating the usability of SORD in populations beyond young adults can help determine its broader applicability. Finally, we observed errors in the raw data from 2 participants for SORD and 3 others for activPAL.

In this study, we did not intend to compare or advance the activity recognition models; rather, the goal was to use the best approach for real-world applications of SORD for real-time intervention. The future development of SORD will include exploring other models (eg, ensemble learning).

In conclusion, SORD accurately detected sitting, standing, and walking activities among healthy young adults, and measurement accuracy was excellent compared to direct observation. While the current iteration of SORD displays promising levels of accuracy, it requires more work and real-world testing in an intervention to assess its applicability. Therefore, SORD holds potential for future integration into evidence- and theory-driven, real-time adaptive interventions to promote activity and reduce sedentary time.

## Appendix

Multimedia Appendix 1 Supplemental Figure 1. Confusion matrix for Model 3 classification algorithms. Sitting, standing and walking were included in the model.

Multimedia Appendix 2 Supplemental Figure 2. Confusion matrix for Model 4 classification algorithms. Lying, sitting, reclining , standing and walking were included in the model.

Multimedia Appendix 3 Supplemental Figure 3. Bland-Altman plot comparing seconds of sitting, standing and walking between the direct observation and SORD activity tracker (Model 3).

Multimedia Appendix 4 Supplemental Figure 4. Bland-Altman plot comparing seconds of lying, sitting, reclining, standing and walking between the direct observation and SORD activity tracker (Model 4).

## Abbreviations

LoA: limits of agreement
PA: physical activity
SB: sedentary behavior
SORD: Sedentary behaviOR Detector
