# Supplementary figures and images for: A Bluetooth-Enabled Device for Real-Time Detection of Sitting, Standing, and Walking: Cross-Sectional Validation Study
Source: JMIR Form Res. 2024 Jan 24;8:e47157. doi: 10.2196/47157 (PMC10851128; doi:10.2196/47157)

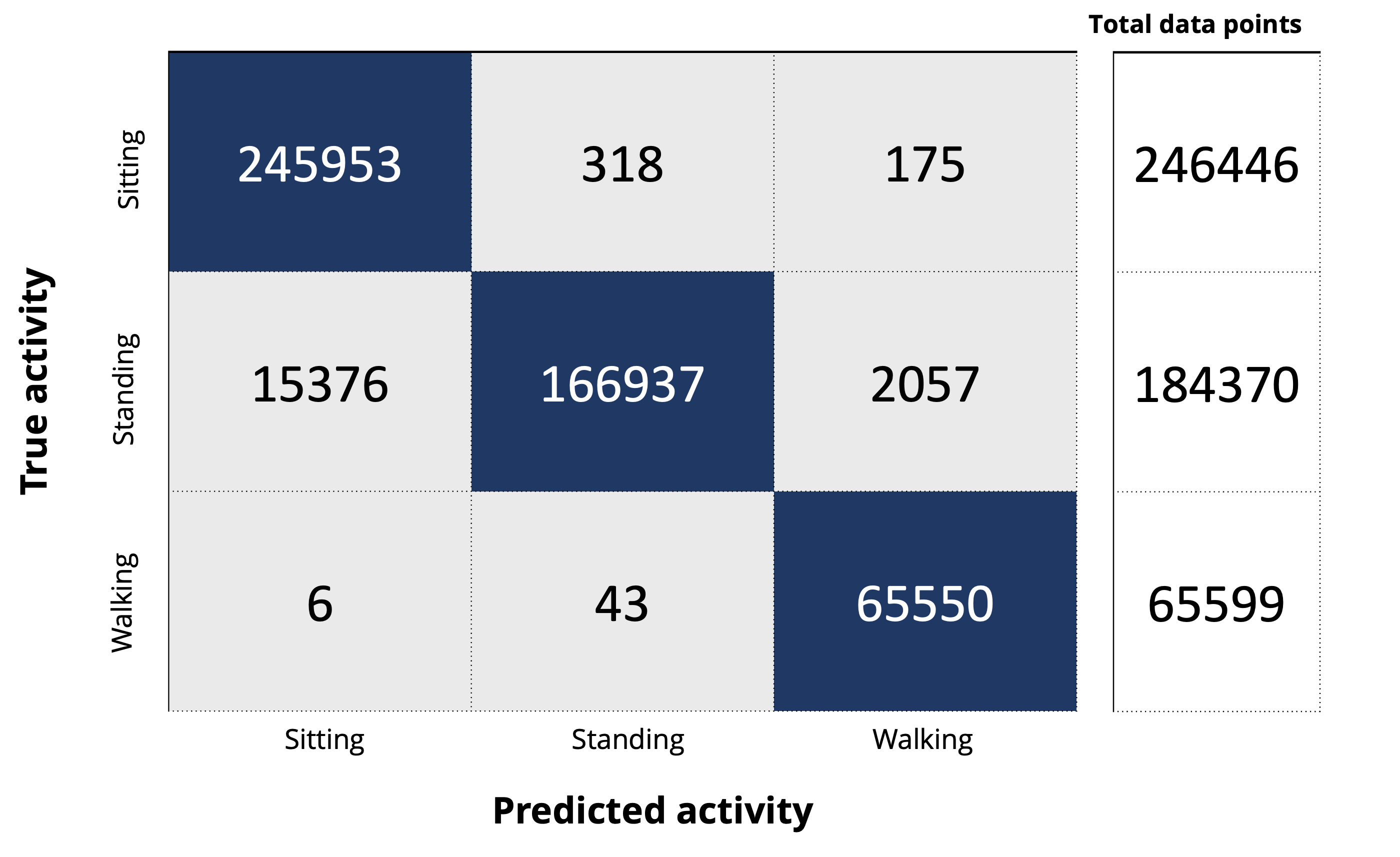

Supplement: Multimedia Appendix 1 [file formative_v8i1e47157_app1.png]

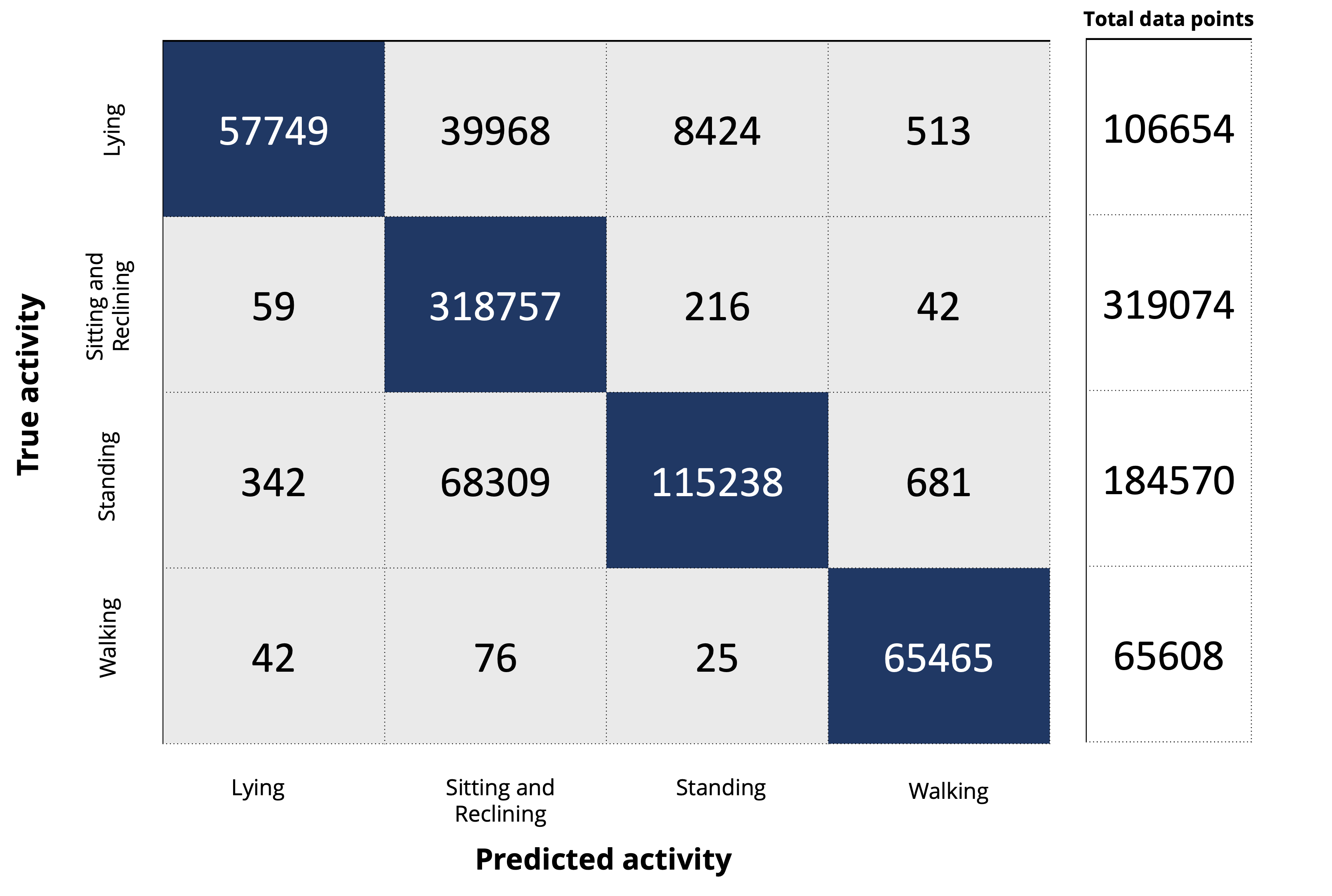

Supplement: Multimedia Appendix 2 [file formative_v8i1e47157_app2.png]

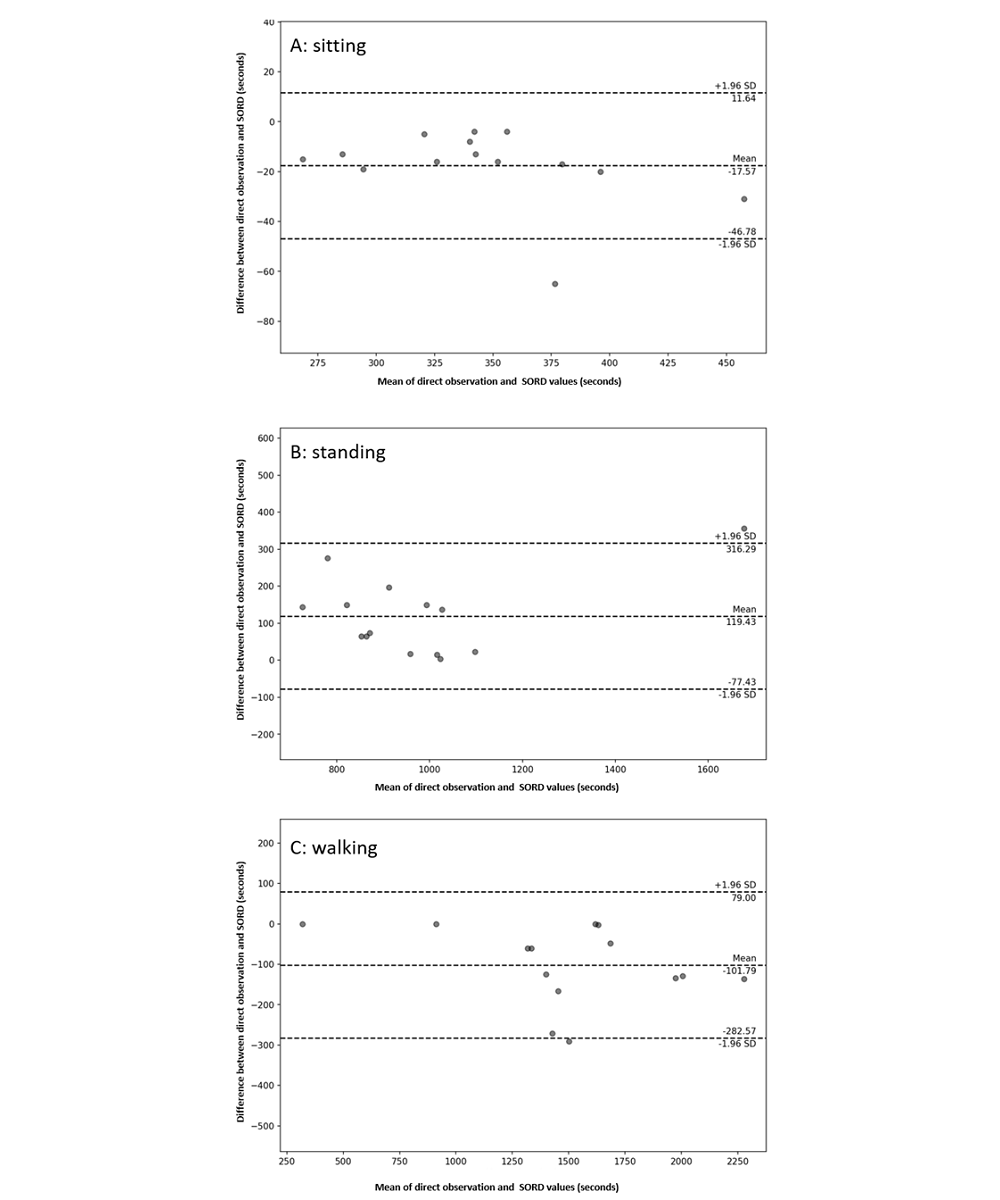

Supplement: Multimedia Appendix 3 [file formative_v8i1e47157_app3.png]

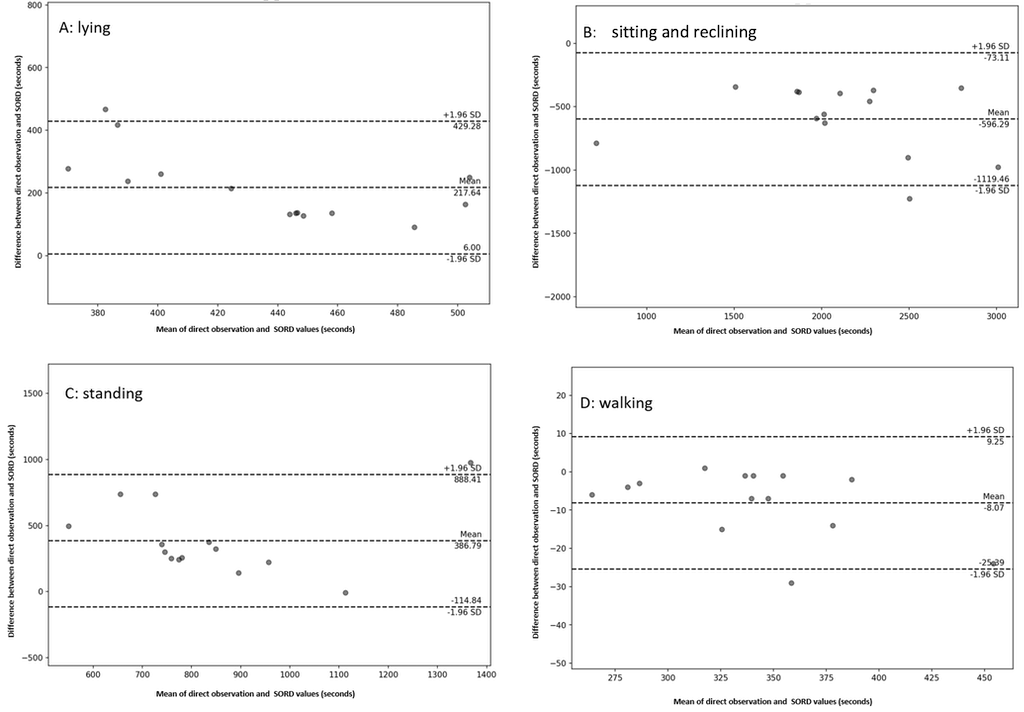

Supplement: Multimedia Appendix 4 [file formative_v8i1e47157_app4.png]
